# Stress-Driven Selective Neuronal Vulnerability in Charcot–Marie–Tooth Disease: From Prodromal Pathology to Therapeutic Implications

**DOI:** 10.3390/cells15030271

**Published:** 2026-01-31

**Authors:** Xianchao Pan, Jiming Xie, Zhiyu Li, Yuemeng Xiang, Yongzhen Yu, Qianqian Cai, Haidong Xu, Ying Wan, Juan Xing

**Affiliations:** 1 Functional Experiment Center, School of Basic Medical Science, Bengbu Medical University, Bengbu 233030, China; panxc@bbmu.edu.cn (X.P.);; 2 School of Basic Medical Science, Southwest Medical University, Luzhou 646000, China; 3 School of Pharmacy, Southwest Medical University, Luzhou 646000, China; 4 Department of Pathophysiology, School of Basic Medical Science, Bengbu Medical University, Bengbu 233030, China; 5 Key Laboratory of Basic and Clinical Cardiovascular Diseases, Bengbu Medical University, Bengbu 233030, China

**Keywords:** Charcot–Marie–Tooth, HSPB1, peripheral neuron, neurodegeneration, stress, neural homeostasis

## Abstract

**Highlights:**

**What are the main findings?**

- HSPB1-related Charcot–Marie–Tooth (CMT) disease is reframed as a gene–environment interaction disorder, where the unique anatomical and metabolic constraints of the peripheral nervous system predispose it to homeostatic failure.
- Selective neuronal vulnerability arises from the convergence of three interconnected pathological axes (proteostatic disturbance, cytoskeletal dysregulation, and mitochondrial dysfunction), which collectively impair stress adaptability.

**What are the implications of the main findings?**

- The findings advocate for a paradigm shift towards early, pre-symptomatic (prodromal) therapeutic intervention, aiming to modulate the HSPB1 interactome and remodel neural homeostasis to forestall degeneration.
- They underscore the necessity for precision medicine strategies that target the underlying maladaptive stress response, moving beyond symptomatic management to achieve true neuroprotection in CMT.

**Abstract:**

Charcot–Marie–Tooth (CMT) disease represents the most prevalent inherited peripheral neuropathy with a broad range of clinical manifestations, inheritance patterns, and causative genes. The primary pathological hallmark is progressive degeneration, predominantly affecting sensory and motor neurons, leading to prominent sensory deficits and progressive motor impairments. While neuropathy-causing mutations in the ubiquitously expressed small heat shock protein HSPB1 account for a subset of axonal CMT cases, the mechanisms underlying the selective vulnerability of peripheral neurons remain poorly understood. In this review, we synthesize emerging evidence to reframe HSPB1-related CMT as a prototypical gene–environment interaction disorder. The unique anatomical exposure and high metabolic demands of the peripheral nervous system (PNS) render it particularly vulnerable to HSPB1 mutation-mediated homeostatic collapse, which manifests through three interconnected pathological axes: proteostatic disturbance, cytoskeletal dysregulation, and mitochondrial dysfunction. Crucially, these deficits converge to impair the stress adaptability of peripheral neurons, creating a maladaptive feedback loop wherein environmental stressors exacerbate intrinsic vulnerabilities. We further propose a phase-specific therapeutic framework that prioritizes early intervention during the clinically silent yet biologically active prodromal stage, when targeted modulation of the HSPB1 chaperone interactome and remodeling neural homeostasis may forestall neurodegeneration. This therapeutic paradigm shift from symptomatic management to preclinical neuroprotection underscores the imperative for precision medicine approaches in future CMT intervention.

## 1. Introduction

Charcot–Marie–Tooth disease (CMT), also referred to as hereditary motor and sensory neuropathy (HMSN), is a group of the most common inherited peripheral neuropathies, with an estimated prevalence rate of up to 40 cases per 100,000 individuals. The disease is also labeled as distal hereditary motor neuropathies (dHMN) when the motor neurons are exclusively affected [1]. The clinical hallmark of CMT is typically a length-dependent motor and sensory neuropathy characterized by progressive muscle weakness and atrophy in the extremities, distal sensory loss, disappearance of tendon reflex, and foot deformities such as pes cavus [2]. CMT patients often experience a physically handicapped process, which is slow and extremely miserable, significantly impacting their quality of life [3]. According to differences in motor nerve conduction velocities (NCVs), CMT neuropathies fall into three subgroups: demyelinating type CMT1 with myelinopathy, in which NCVs are decreased, the axonal type CMT2 with axonopathy, in which NCVs are normal but amplitudes of sensory as well as compound muscle action potentials are decreased, and an intermediate form that exhibits overlapping electrophysiological and neuropathological features of both CMT1 and CMT2 [4]. Historically, CMT exhibits high phenotypic and genetic heterogeneity as well as considerable variability in the onset age, severity, and symptoms. The breakthrough of next-generation sequencing (NGS) technology has driven the identification of more than 130 CMT-associated genes over the past few decades [5]. However, the underlying neuropathogenic molecular mechanisms/pathways are complex and remain enigmatic, which poses a significant challenge in developing therapeutic treatments.

Since 2004, dominant mutations in the gene encoding HSPB1 (heat shock protein family B [small] member 1), also known as HSP27 in humans, have been identified as a pathogenic cause of axonal CMT disease type 2F and dHMN type 2B. HSPB1 is a ubiquitously expressed molecular chaperone belonging to the small heat shock protein (sHSP) family that constitutes a critical element of the protein quality control (PQC) network [6]. This capacity to safeguard proteostasis enables HSPs to act as a highly effective and well-preserved cellular defense system in neurons, exemplified by their role in maintaining α-synuclein homeostasis in Parkinson’s disease [7]. To date, over 30 mutations in HSPB1 have been linked to CMT2 [8,9], whose disease mechanism is multifactorial and involves proteostasis and organelle dysfunction characterized by enhanced chaperone activity, impaired autophagic flux, and mitochondrial defects [10,11,12]. Paradoxically, a central unresolved question is why these ubiquitously expressed mutants selectively target the peripheral neurons while others are resilient in CMT disease. Elucidating the mechanisms underlying selective neuronal vulnerability represents a critical step toward deciphering early pathogenic pathways, which may facilitate the development of predictive biomarkers, prophylactic interventions, and novel therapeutics to halt disease progression.

Given the well-established roles of HSPB1 in cellular homeostasis and stress response, we posit that the selective vulnerability of peripheral neurons in CMT may involve an intrinsic disorder of neuronal homeostasis mediated by HSPB1 dysfunction, and consequently compromised stress adaptability, aggravated by advancing age. This review attempts to provide an interpretation for the pathogenesis of CMT from the perspective of the synergetic effect of stress and genetic mutations. We focus on the roles of stress-boosted selective vulnerability of peripheral neurons in the etiology of axonal CMT subtype, i.e., on how the interplay between environmental stressors and HSPB1 mutation-induced homeostatic disorder promotes progressive peripheral neuropathies, with an emphasis on the mechanistic basis of neural homeostasis breakdown under stress conditions. These findings have important implications for the therapeutic development of CMT disease.

## 2. Contribution of Environmental Stressors to Selective Vulnerability in CMT Neuropathies

Cellular systems in living organisms operate dynamically [13], with their functions exquisitely modulated by microenvironmental cues, as demonstrated by their adaptive response to fluctuating conditions. When confronted with extreme physicochemical or metabolic challenges, cells enter a state with heightened strain and tension termed cellular stress [14], a significant risk factor for cell survival that necessitates robust homeostatic countermeasures. To mitigate stress-induced damage, cells activate evolutionarily conserved defense programs involving proteostatic buffering, redox regulation, and damage repair pathways [15]. Critically, when stress severity surpasses cellular adaptive capacity, these systems fail, triggering irreversible proteotoxicity, organelle dysfunction, and ultimately apoptotic or necrotic demise [16]. The fate of stressed cells thus hinges on both stressor intensity and the cell’s intrinsic stress protection systems.

**Figure 1:**
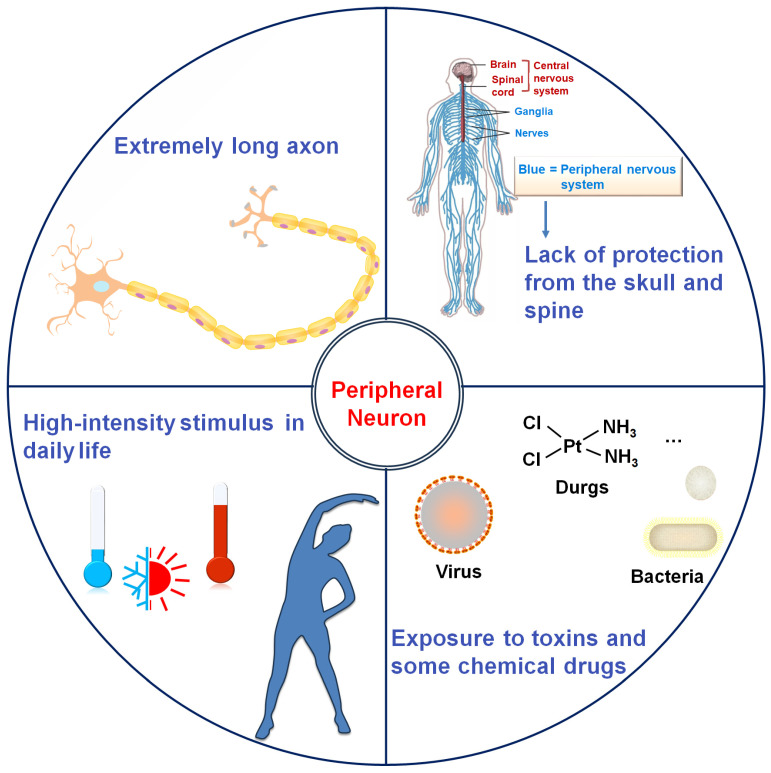
Peripheral neurons are particularly vulnerable to stress due to a confluence of anatomical, environmental, and physiological factors. The schematic of neuron distribution was downloaded from a website (https://www.sohu.com/a/251546452_234786, accessed on 29 January 2026). Created using Microsoft PowerPoint 2016 and Scienceslides 2016 toolset.

Neurons are highly polarized cells characterized by long axons and extensive dendritic arbors, which are essential for their signaling function but also render them structurally vulnerable. This susceptibility is particularly pronounced in peripheral neurons, due to a confluence of anatomical, environmental, and physiological factors (Figure 1). Beyond their intricate morphology, peripheral nerves lack the protective bony encasement afforded to the central nervous system (CNS) by the skull and vertebral column. This exposes them to direct mechanical trauma, compression, and repetitive stress. Furthermore, their terminal projections are ubiquitously distributed in somatic and visceral tissues, placing them in direct contact with potentially damaging environmental stimuli encountered in daily life, such as extreme temperatures (cold or heat) and intense physical exertion [17,18]. A critical vulnerability also arises from their relative lack of a stringent protective barrier. Unlike the CNS, which is shielded by the highly selective blood-brain barrier, the peripheral nervous system (PNS) is guarded by a more permeable blood-nerve barrier. This diminished shielding increases the risk of exposure to systemic toxins (e.g., most viruses and bacteria). Additionally, accumulation of inflammatory mediators (e.g., nuclear factor-κB) and metabolic waste products in peripheral neurons culminates in a heightened propensity for toxic neuropathy and immune-mediated damage [19]. Notably, the PNS retains superior regenerative capacity compared to the CNS [20,21], indicating an evolutionary adaptation to stress resilience. Unsurprisingly, erosion of this stress-adaptive potential precipitates progressive peripheral neurodegeneration.

The knock-in model with mutant HSPB1 expression at physiological levels demonstrated an inability to induce peripheral neuropathy within the murine lifespan, suggesting that the genetic mutations alone are insufficient to fully recapitulate CMT2 phenotypes [22]. This observation aligns with the broader paradigm of selective neuronal vulnerability in neurodegenerative disorders, including Alzheimer’s, Parkinson’s, Huntington’s, and amyotrophic lateral sclerosis (ALS) [23]. The inherent complexity of neurodegenerative disorders has led to an emerging consensus that most, if not all, arise from the interplay between genetic susceptibility and environmental stressors. Saxena and colleagues [24] proposed a stressor-threshold model, wherein age-related accumulation of cellular stress and disease-related misfolding proteins synergistically overwhelm the resilience of particular vulnerable neurons. Emerging evidence has implicated that peripheral neuropathies are modulated by a variety of environmental factors, including heat, exercise-induced oxidative stress, physicochemical insults (e.g., chemotherapy, toxins, and radiation) and pathophysiological stresses (such as hyperthermia, nutrition deficiency, and hypoxia) [25,26]. An extensive body of work has firmly established the genetic linkage between HSPB1 mutations and CMT pathogenesis. Yet, current mechanistic investigations remain disproportionately focused on mutation-induced protein dysfunction, while critically overlooking the potential modulatory effects of environmental stressors, creating a knowledge gap that may substantially limit our comprehensive understanding of disease etiology.

## 3. Neuroprotection Roles of HSPB1 Under Stress Conditions

HSPB1 is a stress-responsive molecular chaperone that mediates adaptive response to diverse stressors such as heat shock, oxidative stress, cytotoxic drugs, apoptosis inducers, and mechanical force [27,28]. It orchestrates multifaceted cytoprotective mechanisms to ensure cell survival, including regulation of the redox environment, prevention of apoptosis, and maintenance of the cytoskeletal dynamics. Neurons exhibit particular reliance on HSPB1-mediated neuroprotection, evidenced by its elevated expression and dynamic redistribution following axonal injury to promote neuronal survival [29,30,31]. While classically recognized for maintaining proteostasis [32], HSPB1 also directly modulates cytoskeletal remodeling and axonal regeneration through interactions with cytoskeletal proteins [33,34]. Recent work further revealed its mitochondrial intermembrane space localization, where it safeguards organellar function during stress [10]. Collectively, HSPB1 integrates proteostatic, cytoskeletal, and bioenergetic regulation to maintain neural homeostasis under stress conditions. This triad of functions not only protects the proteome against stress conditions and enables axonal transport and stress-responsive morphological plasticity, but also preserves mitochondrial function for metabolic recovery post-stress. Such integrative capacity renders HSPB1 indispensable for neuronal stress adaptation. Consequently, HSPB1 mutations may disrupt this homeostatic balance, lowering the stressor threshold for irreversible damage and conferring pathological vulnerability in peripheral neurons.

With the significant neuroprotective role of HSPB1, as well as the recurrent stress endured by peripheral neurons resulting from human movement and environmental factors, we proposed that chronic stress acts as a key driver of peripheral neurodegeneration in HSPB1-mutant CMT disease [35]. Pathologically, HSPB1 mutations trigger a triad of interconnected deficits, including proteostatic collapse, cytoskeletal network abnormality, and mitochondrial dysfunction, collectively eroding neuronal stress adaptability. These disruptions converge to lower the threshold for stress-induced damage, with neural homeostasis breakdown serving as the central pathogenic nexus. In the following, we systematically dissect how environmental stressors interface with HSPB1-dependent homeostatic failure to precipitate the axonopathy characteristic of CMT disease.

## 4. Disturbed Neural Proteostasis

### 4.1. The Role of HSPB1 in the Proteostasis Network

The pathological deposition of misfolded proteins compromises proteome integrity and jeopardizes cell viability [36], which has been identified as a defining hallmark of age-associated neurodegenerative disorders [37,38,39,40]. Environmental stressors can induce protein unfolding and misfolding through conformational destabilization of both nascent and mature polypeptides. Age-related decline in proteostatic regulation exacerbates this process, permitting progressive aggregation of misfolded species [38]. To protect the integrity of the proteostasis network against stress conditions, the cell employs a powerful protein quality control (PQC) system, comprising molecular chaperones coupled with the ubiquitin-proteasome system (UPS) and autophagy-lysosomal degradation pathway [41]. These highly regulated systems operate synergistically to recognize the aggregation-prone unfolded and misfolded proteins, facilitating either their refolding or degradation in response to stress conditions. Failure of this surveillance machinery thus disrupts the proteostatic balance, ultimately leading to cellular dysfunction and death.

HSPB1 is a key player in the PQC system, serving as a sentinel of misfolded proteins, especially under stress conditions [41]. Cellular exposure to environmental stressors elevates the expression of this chaperone, which sequesters and stabilizes stress-induced misfolded proteins in near-native conformation for subsequent degradation by the proteasome molecular machinery. Alternatively, the sequestered substrates by HSPB1 can be released by HSP70/HSP100 for refolding, relying on ATP-dependent chaperones such as HSP60, HSP70, and HSP90 [42]. Hence, the abnormal activity or expression of HSPB1 has been implicated in many diseases, including cancers, neurodegeneration, and neuropathies such as CMT disease.

### 4.2. Structural and Dynamic Features of HSPB1

**Figure 2:**
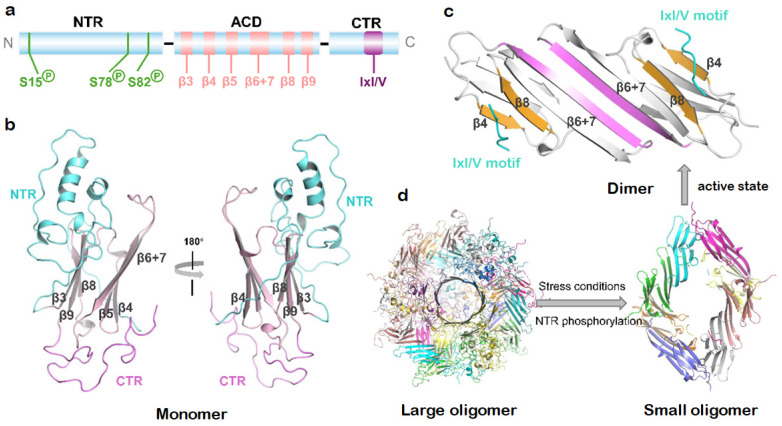
Structure and dynamics of HSPB1. (**a**) Domain architecture of human HSPB1. The phosphorylation sites in the NTR, β-strands in ACD, and the conserved IxI/V motif in the CTR are indicated. N-terminal region (NTR), α-crystallin domain (ACD), C-terminal region (CTR). (**b**) Three-dimensional monomer structure of full-length HSPB1 modeled with the SWISS-MODEL server (https://swissmodel.expasy.org/ (accessed on 15 February 2025)). The NTR (cyan), β-strands in ACD (pink), and CTR (violet) are shown as cartoons. (**c**) Three-dimensional structure of the ACD dimer (white, PDB: 4mjh) bound to the IxI/V peptide (cyan). The dimer interface formed by β6 + 7 strands and β4/β8 groove is indicated. (**d**) Depolymerization of oligomeric HSPB1 mediated by the NTR phosphorylation under stress conditions.

In order to understand the pathogenic mechanism of HSPB1 mutations, it is crucial to decipher the structural determinants for client and co-chaperone interactions. HSPB1 shares the conserved sHSP architecture, featuring a central α-crystallin domain (ACD) flanked by flexible N- and C-terminal regions (NTR/CTR) that orchestrate client binding and oligomeric plasticity [43] (Figure 2a). The ACD adopts an immunoglobulin-like fold with six β-strands assembled into two β-sheets (Figure 2b). Anti-parallel dimerization occurs through pairwise association of β6+7 strands (Figure 2c), with these dimers subsequently assembled into polydisperse homo-oligomers via multiple weak interactions predominantly between NTR/CTR and the ACD dimer (Figure 2d) [44]. The most extensively characterized of such oligomer-stabilizing contacts is the knob-into-hole interaction between the CTR’s IxI/V motif (^179^ITIPV^183^) and the ACD’s β4/β8 groove, which is a versatile binding site mediating both intra- and intermolecular associations. This interaction also facilitates hetero-oligomer formation with other sHSP members and the HSP70 co-chaperone Bcl-2-associated anthanogene-3 (BAG3) that contains IxI/V-like motifs. The intrinsically disordered NTR engages in flexible intermolecular contacts essential for oligomer formation [45,46]. The fleeting NTR-β4/β8 groove contacts regulate chaperone activity, as their disruption liberates the NTR and enhances client binding capacity. While the conserved ACD groove directly engages aggregation-prone substrates, the NTR is essential for full chaperone function, indicating cooperative roles in client recognition [45,47].

Within cells, HSPB1 dynamically interconverts between oligomeric and dimeric states in a concentration-dependent manner [48]. As shown in Figure 2d, cellular stress triggers a phosphorylation-related structural reorganization of HSPB1 [12], primarily involving MAPKAP kinase 2-mediated phosphorylation of NTR residues Ser15, Ser78, and Ser82, which drives the dissociation of larger oligomers into smaller species [44,49]. Biochemical evidence suggests that HSPB1 dimers represent the active species capable of binding misfolded client proteins [48,50], with the monomer/dimer ratio critically regulating chaperon activity [51]. While technical challenges persist in determining the native molecular size of HSPB1 in vivo, particularly in distinguishing between self-oligomerization and client protein interactions [12], the phosphorylation-associated structural plasticity unequivocally underlies its capacity to manage misfolded proteins.

### 4.3. Proteostasis Dysfunction Caused by Mutant HSPB1

#### 4.3.1. Altered Chaperone Activity

The majority of currently discovered CMT-associated HSPB1mutations are located in the ACD, with additional variants occurring in the NTR and CTR. The pathogenic mechanism is mutation-dependent. Nonsense and frameshift mutations commonly generate premature termination codons, leading to the production of truncated protein isoforms [52]. In patient-derived primary fibroblasts, truncated HSPB1 (particularly the M169fs variant) has been shown to interact with the wild-type, which significantly weakens the cellular capacity to manage stress-induced misfolded proteins [53].

Under physiological conditions, wild-type HSPB1 exhibits an intrinsic propensity for self-association, forming dynamic polydisperse oligomers [54]. Several mutations in the ACD (R127W, S135F, R136W) promote oligomer-to-monomer transitions [51], allowing for enhanced binding of HSPB1 to client proteins such as SQSTM1 [11]. The hyperactivation enables these mutants to interact with non-native substrates, including tubulin [51]. Such aberrant interactions disturb both stress-responsive substrate binding and the physiological function of peripheral neuron maintenance proteins. CTR mutations frequently stabilize oversized oligomers at the expense of chaperone activity. For instance, Pro182 mutations within the IxI/V motif (^179^ITIPV^183^) induce pathological homo-oligomer formation [55,56]. NMR experiments reveal that the P182L mutation weakens IxI/V-β4/β8 groove interactions, leaving this binding site more accessible for other IxI/V-containing proteins like HSP70 and cochaperone BAG3 [55]. Meanwhile, the CTR dissociation strengthens NTR-ACD contacts, consequently altering oligomeric architecture and chaperone activity [44]. Additional CTR variants (P182S, P182A, S187L) form exceptionally stable oligomers resistant to concentration-dependent dissociation, often correlating with more severe clinical phenotypes [55,56,57]. NTR mutations typically disrupt stress-responsive phosphorylation, thus locking HSPB1 in the oligomeric state [58]. Together, these mutations break the physiological oligomer–dimer–monomer equilibrium through either excessive monomerization or pathological oligomer stabilization, severely compromising stress-responsive proteostasis. Notably, the biochemical properties of substituted residues also influence HSPB1’s chaperone activity [52]. For example, the CTR variants (T164A, T180I) decrease thermal stability and induce abnormal clustering, while R188W impairs the chaperone activity through perturbations in CTR flexibility and polarity [56].

The stress-induced formation of tight HSPB1-HSPB6 hetero-oligomers through interactions mediated by the HSPB1’s NTR is essential for its chaperone function [46,59]. NTR mutations (G34R, P39L, E41K) significantly attenuate both the kinetics and extent of MAPKAP kinase 2-dependent HSPB1 phosphorylation, particularly during early-stage catalysis (<5 h) [60]. This phosphorylation deficit consequently impedes the dissociation of HSPB1 homo-oligomers, disrupting subsequent hetero-oligomer assembly and thereby diminishing chaperone activity. While the G84R mutation preserves phosphorylation capacity, it decreases NTR mobility and accessibility, thus weakening its interaction with HSPB6 for the hetero-oligomer formation [49]. Furthermore, ACD mutations (L99M, R140G, K141Q) affect the overall stability of the ACD dimer and weaken the HSPB1-HSPB6 interactions critical for hetero-oligomerization [49,61].

Collectively, HSPB1 mutation regulates its chaperone activity through affecting protein stability, self-aggregation and abnormal interaction with other heat shock proteins and non-native substrates. Most critically, mutation-induced impairment of HSPB1’s capacity to recognize and compartmentalize misfolded proteins, particularly during proteotoxic stress, results in catastrophic failure of protein quality control.

#### 4.3.2. Impaired Autophagic Degradation

The ubiquitin-proteasome system (UPS) serves as the primary degradation pathway for chaperone-escorted misfolded proteins [41]. When the chaperones and UPS system are broken or overwhelmed, misfolded proteins escape surveillance and form cytotoxic soluble aggregates. To counteract this threat, cells initiate autophagy-lysosomal degradation, wherein misfolded proteins are sequestered within autophagosomes that undergo microtubule-mediated perinuclear trafficking for lysosomal fusion [62]. Furthermore, the small aggregates can be transported directly to the microtubule organization center to form perinuclear aggresomes, enabling their subsequent macroautophagic clearance [63].

Emerging evidence implicates HSPB1 mutations in autophagy dysfunction. Pathogenic ACD variants (R127W, S135F) exhibit enhanced binding to the autophagy receptor SQSTM1, impairing autophagosome assembly under starvation [11]. The P182L mutation also disrupts stress-induced autophagy, exacerbating mitochondrial fragmentation and axonal degeneration [64]. Our recent work demonstrates that the abnormal binding of the S135F variant to α-tubulin blocked microtubule-dependent axonal transport, leading to the accumulation of misfolded proteins in stressed cells [35]. These findings collectively suggest that it interferes with neuronal proteostasis through aberrant interactions with autophagy machinery components. Recent studies reveal HSPB1’s additional role in lysophagy, where its phosphorylation-dependent recruitment to damaged lysosomes facilitates their selective autophagic turnover [65]. This raises an intriguing question regarding whether NTR mutations that affect HSPB1 phosphorylation might impair the lysosomal quality control. Further investigations are needed to fully elucidate the impact of mutant HPSB1on lysosomal homeostasis.

Proteostasis maintenance becomes particularly crucial under cellular stress conditions. This requirement is especially pronounced in post-mitotic neurons, which must maintain functional integrity throughout an organism’s lifespan through coordinated chaperone networks and selective degradation pathways, including macroautophagy. Prominently, peripheral neurons face additional proteostatic challenges from environmental stressors that upregulate HSPB1 expression for cellular protection. CMT-causative HSPB1 mutations exhibit aberrant chaperon activity (Table 1) and, more critically, disrupt functional interactions with key degradation machinery components (i.e., HSP70, BAG3, SQSTM1, α-tubulin). These pathological interactions compromise misfolded protein clearance, collapsing the PQC system in stressed peripheral neurons. The consequent proteostatic failure ultimately drives the selective vulnerability characteristic of peripheral neuropathy.

## 5. Dysregulated Cytoskeletal Network

The functional integrity of peripheral neurons relies on their elongated axonal projections, whose polarized architecture is maintained by a highly organized cytoskeletal framework comprising microtubules (MTs), neurofilaments (NFs) and actin microfilaments. This tripartite cytoskeletal system not only provides structural support and enables intracellular transport, but also mediates dynamic remodeling and mechanical responses in neurons [71,72]. The exceptional length of peripheral axons and their permanent exposure to environmental stressors necessitate remarkable cytoskeletal plasticity to preserve functionality across diverse physiological conditions.

### 5.1. Acetylation-Related Biphasic Alteration of MT Dynamics

The structural integrity of MT network is essential for maintaining axonal polarity and facilitating bidirectional cargo transport [73]. Anterograde transport, mediated by kinesin motor proteins, delivers newly synthesized proteins and lipids to the distal synapses to sustain neuronal activity. Conversely, retrograde transport, driven by the dynein-dynactin motor complex, returns aged cellular materials (e.g., misfolded proteins produced in distal axons) to the soma for degradation and recycling [74]. Additionally, retrograde transport conveys intracellular signals from distal axons to the soma, allowing neurons to respond to environmental cues [75]. Given the extraordinary length of axons, peripheral neurons are particularly vulnerable to disruptions in MT stability and axonal transport.

The MT network is a dense, highly dynamic cytoskeletal scaffold composed of α/β-tubulin heterodimers. Tubulin acetylation is a critical modification conferring MT flexibility and mechanical resistance [76], particularly for long-lived MTs [77]. The loss of this post-translational modification predisposes MTs to stress-induced damage. Pathogenic HSPB1 mutations induce time-dependent MT network disruption. Transgenic S135F/P182L mice developed CMT-like neuropathy at 6 months, showing significant tubulin deacetylation in sciatic nerves [78]. Intriguingly, presymptomatic (3-month-old) mice exhibited enhanced HSPB1^S135F^ chaperone activity and abnormal α-tubulin binding in dorsal root ganglia (DRG) neurons without acetylation defects [79]. This phenotype was recapitulated in HEK293 cells expressing HSPB1^R127W/R136W^, where MTs exhibited cold-resistant stabilization that paradoxically compromises cellular stress adaptation.

To explain the biphasic alteration of MT dynamics in HSPB1 mutant mice, Almeida-Souza et al. [80] proposed a temporal model, in which mutant HSPB1 initially hyperstabilizes MTs via aberrant tubulin interactions, and then triggers compensatory tubulin deacetylation as a feedback mechanism to restore MT dynamics, ultimately resulting in excessive tubulin deacetylation and consequent MT network destabilization. Environmental stress accelerates this cascade, as evidenced by the preserved α-tubulin acetylation levels in the spinal cord of HSPB1^S135F^-expressed mice [81], a protected site from environmental stress compared to peripheral nerves. These findings mandate longitudinal or stress-challenge experimental designs to capture HSPB1 mutation-caused MT pathology, which may explain negative results in primary motor neurons with HSPB1 mutants (P39L, R140G, and S135F) [82]. The biphasic transition also underscores that early MT hyperstabilization may represent a latent, compensatory phase of dysfunction, whereas subsequent destabilization marks the onset of irreversible axonal transport failure and clinical symptom manifestation.

### 5.2. Multimodal Disruption of MT-Dependent Axonal Transport

Beyond the effect on MT dynamics, pathogenic HSPB1 variants compromise axonal transport through multiple mechanisms [78]. The primary pathway involves dysregulation of α-tubulin acetylation—a critical post-translational modification that orchestrates motor protein navigation along axonal MTs. Certain cargoes (e.g., mitochondria) demonstrate preferential binding to acetylated MT tracks [82,83]. Symptomatic HSPB1^S135F^-expressed mice showed reduced levels of α-tubulin acetylation and impaired mitochondrial motility in DRG neurons. Pharmacological HDAC6 inhibition rescues this transport deficit [78], confirming the mechanistic link between acetylation status and cargo mobility. A secondary, acetylation-independent mechanism emerges from aberrant HSPB1/α-tubulin interactions [79]. Our recent findings reveal that HSPB1^S135F^/α-tubulin interactions specifically block the retrograde transport of stress-denatured proteins to perinuclear quality control compartments [35], leading to neurotoxic protein accumulation during proteostatic challenge. This phenotype mirrors the MT-related pathology in peripheral neuropathy.

Axonal transport deficit caused by HSPB1 mutations extends to motor protein dysfunction through pathological sequestration. In HSPB1^P182L^-expressing cells, mutant HSPB1-induced protein aggregates physically capture p150 [84], the critical component of the dynein-dynactin complex. This depletion of functional p150 from axonal and synaptic compartments disrupts the retrograde transport of growth factors and signaling molecules for neuronal survival, establishing a vicious cycle: impaired transport begets aggregate accumulation, which further depletes motor proteins. This self-amplifying cascade ultimately drives the selective vulnerability of peripheral neurons.

### 5.3. Mutation-Dependent NF Dysfunction

Another cytoskeletal vulnerability in HSPB1-causative disorders is related to neurofilaments (NFs)—neuron-specific heteropolymers of NF-L, NF-M, and NF-H subunits [85]. Synthesized somatically, NFs undergo kinesin-mediated anterograde and dynein-driven retrograde transport along MTs. As the most abundant cytoskeletal components in peripheral axons, NFs structurally support myelinated axons while regulating radial growth to maintain optimal axonal diameter and conduction velocity [86,87]. Structurally, NF subunits assemble into 10 nm filaments with α-helical core domains and projecting side-arms [88], whose functional integrity depends on regulated post-translational phosphorylation [89]. NF-L mutations in CMT2E exemplify the catastrophic consequences of NF assembly defects, leading to severe axonal degeneration [90].

Neurofilament aggregation caused by mutations in genes that encode NFs or regulate their metabolism has been discovered in neurodegeneration [91]. HSPB1 safeguards the NF network by preventing stress-induced NF protein aggregation through co-localizing with NFs and suppressing non-covalent interactions between NFs [92]. Pathogenic HSPB1 mutations subvert this protective function. Zhai et al. [93] demonstrated that the S135F variant exacerbates NF-L aggregation and disrupts filament networks in motor neurons. NF-L deletion abolished S135F-induced neurotoxicity, directly implicating NF dysregulation in CMT2F pathogenesis. Additionally, Evgrafov et al. [8] observed perinuclear accumulation of NF-L aggregates in HSPB1^S135F^-expressing cells, suggesting their anterograde transport impairment. Subsequently, Holmgren and colleagues documented reduced anterograde NF-M movement and enhanced retrograde transport in SH-SY5Y cells expressing S135F/P182L variants [94], attributed to Cdk5-mediated hyperphosphorylation of NFs disrupting their interaction with kinesin [88]. Similar hyperphosphorylation was observed in SH-SY5Y cells expressing P7S/G53D/Q128R variants; however, neither NF aggregates nor altered NF-H staining patterns were detected [52]. This aligns with the observation of Kalmar et al. [81], who detected no NF-L alterations in mutant (S135F, P39L, and R140G) HSPB1-transduced primary motor neurons. This phenotypic discrepancy in NF aggregations suggests mutation-specific effects on the NF network and expression-level related mutant chaperone activity.

### 5.4. Defects in Actin Dynamics

During postnatal development, peripheral axon elongation accommodates body growth [95], yet 60% of HSPB1 mutation carriers exhibit length-dependent neuropathy with initial lower limb selectivity [5]. This clinical phenotype implicates that mechanical stress resulting from ambulation and physical activity may accelerate peripheral axon degeneration. Axonal regeneration following mechanical injury is critically dependent on growth cones, where intrinsic mechanical forces directly promote axonal extension [96,97]. In force-generating axonal growth cones, the leading edge protrusion is primarily driven by actin dynamics [98], where globular actin (G-actin) polymerizes into filamentous actin (F-actin), generating the tension necessary for membrane protrusion and neurite extension [99]. Disruptions in actin dynamics and lamellipodia formation are implicated in CMT2B pathogenesis, a dominant-intermediate form caused by dynamin mutations [100].

As a key regulator of actin dynamics, phosphorylated HSPB1 modulates stress-responsive actin cytoskeleton remodeling in non-neuronal cells [34,101], while neuronal HSPB1 interacts with actin to facilitate focal adhesion formation, pseudopod extension, neuritogenesis, and neurite outgrowth [33]. Notably, HSPB1 overexpression in cortical neurons counteracts NogoA-mediated inhibition of neurite elongation, suggesting its importance in overcoming axonal growth barriers [102]. These observations posit HSPB1-actin interactions as crucial for force generation and growth cone dynamics, raising the question of whether CMT-linked HSPB1 mutations compromise this function during axonal elongation.

Collectively, the extreme length of peripheral axons renders them particularly susceptible to cumulative mechanical stresses (e.g., tension, torsion, and compression) [103]. HSPB1 mutations destabilize the cytoskeletal plasticity and essential dynamics required for stress adaptation, leading to defects in axonal transport and regeneration. Thus, cytoskeletal dysregulation becomes a pathogenic cause for the selective degeneration of peripheral neurons in HSPB1-linked CMT neuropathies.

## 6. Mitochondrial Dysfunction

Mitochondria, the principal sites of cellular energy production through oxidative phosphorylation, face extraordinary operational challenges in neurons [104], where synaptic activities distant from the cell body impose relentless energy requirements [105]. This energetic burden escalates dramatically in peripheral neurons, where the extreme axonal length necessitates highly efficient energy metabolism to sustain the bidirectional transport system. The specialized architecture of Schwann cells, with their extensive myelin lamellae enveloping axons, further underscores the critical need for substantial energy investment [106]. Consequently, these unique metabolic demands create a perfect storm that renders peripheral nerves exquisitely sensitive to mitochondrial dysfunction, making them the canaries in the coal mine for metabolic perturbations.

Mitochondria are encircled by a sophisticated double-membrane architecture, i.e., the outer membrane and inner membrane, which create specialized compartments within the organelle. Mitochondria exhibit remarkable plasticity in response to metabolic challenges, undergoing membrane fusion to modulate oxidative phosphorylation capacity during periods of increased energy demand [106]. Such dynamic behavior proves particularly crucial for maintaining mitochondrial function under stress conditions [19]. Growing data has implicated mitochondrial dysfunction in CMT pathogenesis, with documented abnormalities in mitochondrial transport and functionality.

### 6.1. Disrupted Mitochondrial Axonal Transport

The strategic positioning of mitochondria along axons constitutes a fundamental biological solution to neuronal energy challenges. In peripheral nerves, mitochondria show preferential localization at three critical hotpots, including the axon initial segment, distal terminals near neuromuscular junctions in motor neurons or sensory endings, and axonal lesion sites such as demyelinated regions [107]. This precise spatial distribution relies on an intact cytoskeletal network and efficient axonal transport system, where mitochondria undergo bidirectional movement along MTs, with kinesin-1 driving anterograde transport toward synaptic terminals and the dynein-dynactin complex mediating retrograde movement, while stationary mitochondria are primarily anchored to neurofilaments (NF-M and NF-H) [106,108].

Emerging evidence implicates tubulin acetylation defects in the pathogenesis of CMT2A caused by mutations in mitofusin 2 (MFN2), a dynamin-like GTPase that is anchored within the outer mitochondrial membrane [85,109,110]. Importantly, MFN2 mutations disrupt mitochondrial dynamics—including fusion, transport, and adaptive remodeling—which has been indicated as a central pathogenic mechanism driving early axonal dysfunction in CMT2A [111,112]. Building on the role of HSPB1 in cytoskeletal regulation, d’Ydewalle et al. [78] demonstrated that DRG neurons from symptomatic S135F mice showed decreased α-tubulin acetylation and impaired mitochondrial motility. Strikingly, pharmacological inhibition of HDAC6 rescued this defect, thereby ameliorating CMT symptoms. This highlights the significant relationship between mitochondrial dynamics and neuronal survival in CMT pathogenesis. However, mitochondrial transport deficits occur at the advanced disease stage and depend on mutation sites, as evidenced by normal mitochondrial numbers and motility observed from presymptomatic S135F mice and both symptomatic/presymptomatic P182L mice. Furthermore, reduced α-tubulin acetylation and impaired mitochondrial movement are both detected in patient-derived iPSC motor neurons carrying S135F or P182L mutations, revealing cell context-dependent effects on mitochondrial dynamics [113].

### 6.2. Defects in Mitochondrial Function

The pathogenic effects of mutant HSPB1 on mitochondrial homeostasis extend beyond transport defects to encompass a multi-faceted functional collapse. Kalmar et al. [81] reported a significant reduction in retrograde mitochondrial trafficking in primary HSPB1-mutant (S135F, P39L, R140G) motor neurons, while retrograde movement of p75 neurotrophin receptor remained intact, suggesting cargo-specific transport vulnerability. Further investigations revealed that mitochondrial redox imbalance in HSPB1-mutant neurons induces low-level oxidative stress, potentially contributing to heightened mitochondrial vulnerability and transport deficiency. Additionally, the S135F mutant exhibited enhanced binding to ceramide synthases (CerSs) in HT-22 cells, facilitating mitochondrial hyperfusion and thus altering mitochondrial morphology [114]. These changes mirror the elevated mitochondrial density observed in sciatic nerves of 12-month-old R136W mice [105,115], an ACD-domain mutation with enhanced client protein affinity [51]. Notably, the absence of clinical symptoms in 12-month-old R136W mice reveals a critical temporal disconnect between mitochondrial malfunction and clinical CMT symptoms, offering potential therapeutic opportunities [115].

Mitochondrial proteostasis represents a formidable biological challenge, requiring coordinated import of ~1000 nuclear-encoded proteins and stringent quality control via the mitochondrial PQC system [116]. A recent work demonstrated substantial enrichment of HSPB1 in the mitochondrial intermembrane space following heat shock, underscoring the key role of HSPB1 in mitochondrial stress protection [10]. The P182L variant, which correlates with the most severe CMT phenotype, leads to impaired mitochondrial import through the formation of aberrant oligomers. At the same time, the P182L mutation acts dominantly through stoichiometric trapping of wild-type subunits. This dominant-negative mechanism creates a proteostatic crisis: the mutant oligomers sequester available HSPB1 pools, starving mitochondria of functional chaperones and compromising mitochondrial PQC [10]. This mutant HSPB1-induced PQC failure in mitochondria promotes protein aggregation in mitochondria and consequent mitochondrial dysfunction under stress conditions.

## 7. Therapeutic Implications

**Figure 3:**
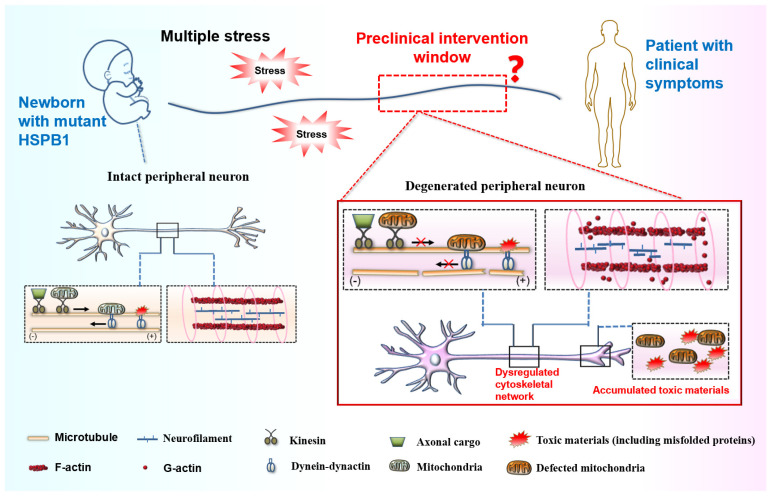
Pathogenesis of selective and progressive peripheral neurodegeneration in CMT disease. Peripheral neurons carrying HSPB1 mutations exhibit increased vulnerability to stress conditions, resulting in selective degeneration of the peripheral nervous system through multiple pathological mechanisms: dysregulated cytoskeletal network, accumulation of toxic materials caused by disturbed neural proteostasis and impaired axonal transport, as well as mitochondrial dysfunction. These homeostatic imbalances mostly precede clinical manifestation, suggesting a preclinical intervention window for developing stage-specific therapeutic strategies in CMT management. Created using Microsoft PowerPoint 2016 and Scienceslides 2016 toolset.

Despite significant improvements in the diagnostic capabilities, no effective targets and curative treatments are currently available for CMT disease. Advances in elucidating the pathogenic mechanism of HSPB1 mutations stimulated efforts to develop therapies for a specific CMT subtype. Recently, a limited number of active compounds have been identified that can correct axonal transport defects or promote autophagosome formation to induce autophagy [64,78]. Despite showing potential in rescuing axonal degeneration, their efficacy and safety require systematic validation and evaluation in clinically relevant translational models. CMT neuropathy is a chronic, hereditary disorder that remains incurable. Emerging evidence suggests that therapeutic intervention during the preclinical stages of neurodegenerative disorders offers substantially greater efficacy in modifying disease progression compared to symptomatic-phase treatments. This highlights the neuroprotective potential of early therapeutic windows for achieving clinically meaningful outcomes. As we have discussed, homeostatic imbalances caused by HSPB1 mutations in stressed peripheral neurons often occur prior to clinical manifestation, advocating for a feasible preclinical intervention window for developing stage-specific therapeutic strategies in CMT management (Figure 3). Thus, identifying reliable biomarkers to accurately define this window is a critical prerequisite for enabling timely and precise pre-symptomatic intervention. Meanwhile, we propose that targeting prodromal pathology and remodeling homeostatic systems to enhance the stress adaptability of peripheral neurons may alleviate disease progression in axonal forms of CMT.

Accumulating research data support mutant HSPB1 as a promising therapeutic target for attenuating the progression of the related CMT subtype. A hallmark pathogenic event during the presymptomatic stage involves autophagy impairment, driven by aberrant interactions between mutant HSPB1 and binding partners such as α-tubulin or SQSTM1/p62. Therefore, we hypothesize that selectively disrupting these pathological protein-protein interactions could restore autophagic flux, promote the clearance of stress-induced misfolded proteins, and improve stress adaptation in peripheral neurons—ultimately attenuating axonal degeneration in CMT neuropathies. Several therapeutic strategies emerge as potential avenues. Small-molecule inhibitors represent one viable option by directly blocking mutation-induced protein interactions and reactivating autophagic pathways under stress conditions. Alternatively, proteolysis-targeting chimeras (PROTACs) offer a novel modality to chemically degrade mutant HSPB1 via the ubiquitin-proteasome system. Recent progress in antisense oligonucleotide (ASO) therapeutics therapy also holds promise for specific CMT subtypes and other neurodegenerative disorders [117,118,119]. Tailored ASOs could either reduce levels of axonal degeneration biomarkers or directly suppress mutant HSPB1 mRNA expression, thereby diminishing the accumulation of pathogenic protein at early disease stages. In parallel, artificial intelligence (AI)-driven approaches now permeate drug discovery pipelines from diagnosis to lead optimization [120], as exemplified by the rational design and mechanistic study of mitofusin agonists (e.g., Chimera C/MiM 111) for CMT2A, which aim to rescue pathogenic mitochondrial dynamics by modulating mitofusin conformation [111]. Beyond drug discovery, these computational methods hold significant potential for identifying dynamic prodromal biomarkers, which are crucial for defining the preclinical intervention window and enabling timely, stage-specific therapy. Returning to HSPB1, such computational and high-throughput strategies are also expected to accelerate the development of therapeutic agents targeting mutant HSPB1 and its pathological interactions for CMT. While these approaches outline compelling directions for future CMT therapeutics, further extensive validation is essential to evaluate their safety and efficacy in robust preclinical models.

## 8. Conclusions and Future Prospects

HSPB1 is a ubiquitously expressed molecular chaperone that has a central role in maintaining neural homeostasis and physiology, yet pathogenic mutations in this small heat-shock protein can cause axonal CMT disease that is characterized by progressive peripheral neuropathy. While recent advances have revealed the molecular and cellular mechanisms underlying the pathogenesis of this neuromuscular disorder, the mechanistic basis for the selective vulnerability of peripheral neurons remains poorly understood. Identifying the driving factors for the progressive and selective neurodegeneration is likely crucial for pinpointing early pathogenic mechanisms and advancing the development of intervention strategies to prevent and treat CMT disease.

Based on the unique anatomical location and physiological demands of the PNS, together with the stress protection role of HSPB1, this review, for the first time, presents a mechanistic framework linking the contribution of chronic environmental stressors to the slowly progressive degeneration of peripheral neurons and provides a novel insight into the etiology of axonal CMT diseases. At the subcellular level, neuropathy-causing mutations in HSPB1 disrupt three critical stress-responsive homeostatic systems, including protein quality control, cytoskeletal network, and mitochondrial dynamics, and thereby significantly reduce cellular stress adaptability, with peripheral neurons showing particular vulnerability due to their sustained exposure to metabolic and environmental challenges. Accordingly, we suggest that HSPB1-mutant CMT could be regarded as a quintessential gene–environment interaction disorder. Furthermore, we propose a phase-specific therapeutic framework that prioritizes early intervention during the clinically silent but biologically active prodromal stage, aiming to undermine the progression of peripheral neurodegeneration.

Notwithstanding these advances, critical challenges persist in developing effective preclinical therapeutic interventions. The field currently grapples with four interconnected obstacles: (i) the lack of rigorous validation of HSPB1 as a therapeutic target, owing to its pleiotropic roles in diverse cellular processes and context-dependent mechanism of action—including recent findings that HSPB1 can be secreted into the extracellular space, mediating non-cell-autonomous chaperone signaling critical for organismal proteostasis [121,122], which may interact with and exacerbate the cell-autonomous vulnerabilities within peripheral neurons; (ii) the absence of consensus in defining the preclinical phase, during which molecular pathology precedes clinical symptoms, particularly with respect to biomarkers that can reliably identify the transition from presymptomatic to symptomatic stages; (iii) the limitation of existing animal models, which often fail to accurately replicate human CMT pathophysiology, especially in aspects such as axonal transport defects and mitochondrial dysfunction, thereby constraining preclinical drug evaluation; and (iv) the substantial clinical heterogeneity in symptom onset, rate of progression, and phenotypic severity, driven by over 100 causative mutations affecting peripheral nerve function, necessitating genotype-specific therapeutic approaches and personalized trial designs to address divergent disease trajectories and treatment responses. Collectively, these challenges underscore urgent requirements for target validation, advanced biomarker discovery, improved model systems with humanized genetic backgrounds, and innovative clinical trial methodologies tailored to the genetically heterogeneous landscape of CMT.

## Figures and Tables

**Table 1:** Disturbed proteostasis caused by dHMN/CMT2-associated mutations in HSPB1.

Mutations	Location	Disease/Inheritance of the Phenotype	Mechanism of Disordered Protein Homeostasis	References
**G34R**	NTR	dHMN Late onset (>50)	High tendency to self-association; Resistant to phosphorylation-induced dissociation; Low chaperon-like activity; Decreased formation of hetero-oligomers.	[58,60,66]
**P39L**	NTR	dHMN/CMT2 (AD) Late onset (>50)	High tendency to self-association; Decreased phosphorylation; Resistant to phosphorylation-induced dissociation; Low chaperon-like activity; Decreased formation of hetero-oligomers.	[58,60,67]
**E41K**	NTR	dHMN (AD) Early onset	High tendency to self-association; Resistant to phosphorylation-induced dissociation; Low chaperon-like activity; Decreased formation of hetero-oligomers;	[58,60,66]
**G53D**	NTR	dHMN (AR)	Hyperphosphorylation of NFs	[52]
L58fs*105	NTR	dHMN (AD)	Proteasomal degradation	[52]
A61fs*100	NTR	dHMN	Proteasomal degradation	[52]
**G84R**	NTR	dHMN/CMT2 (AD)	Decreased accessibility of the NTR; Decreased formation of hetero-oligomers.	[49,67]
**V97L**	ACD	dHMN (AD)	Increased apoptosis under stress (H_2_O_2_)	[57]
**L99M**	ACD (β3)	dHMN/CMT2 (AR)	Decreased formation of hetero-oligomers	[67]
**R127W/R127L**	ACD (β5)	dHMN/CMT (AD)	Increased monomerization; High chaperon-like activity; Increased susceptibility to ER stress; ER stress-induced apoptosis.	[51,68]
**S135F/S135C**	ACD (β7)	dHMN/CMT2 (AD)	Increased monomerization; High chaperon-like activity; Inhibited clearance of misfolded proteins caused by disturbed axonal transport; Impaired macroautophagy; Increased susceptibility to ER stress; ER stress-induced apoptosis.	[11,35,51,68]
**R136W/R136L**	ACD (β7)	CMT2 (AD)	Increased monomerization; High chaperon-like activity; Increased susceptibility to ER stress; ER stress-induced apoptosis.	[51,68]
**T139M**	ACD (β7)	CMT2F (AD)	Increased apoptosis	[69]
**R140G**	ACD (β7)	dHMN/CMT2 (AD)	Decreased formation of hetero-oligomers; Decreased thermal stability; Low chaperon-like activity.	[61,67]
**K141Q**	ACD(β7)	dHMN (AD)	Decreased formation of hetero-oligomers; Decreased thermal stability.	[61]
**T151I**	ACD	dHMN (AD)	Increased susceptibility to ER stress; ER stress-induced apoptosis.	[68]
**T164A**	ACD (β9)	CMT2 (AD)	Destabilization of the quaternary structure; Decreased thermal stability	[56]
M169fs*2	ACD	dHMN	C-terminally truncated protein; Impaired tolerance to unfolded protein stress.	[53]
**T180I**	CTR	CMT2 sporadic	Formation of hydrophobic cluster consisting of three Ile produced a small increase in thermal stability	[56]
**P182L/P182S/P182A**	CTR	dHMN (AD)	Increased formation of metastable large oligomers; Induced formation of mixed oligomers with wild-type HSPB1; Impaired autophagy (P182L).	[56,64,70]
**S187L**	CTR	dHMN de novo	Tendency to form large cytoplasmic aggregates	[52]
**R188W**	CTR	CMT2	Low chaperon-like activity	[56]

## Data Availability

No datasets were generated or analysed during the current study. Data sharing is not applicable to this article.
